# Dissecting the human T and B cell response to pathogens

**DOI:** 10.1186/ar3404

**Published:** 2011-09-16

**Authors:** Antonio Lanzavecchia

**Affiliations:** 1 Institute for Research in Biomedicine, Bellinzona, Switzerland; 2 Institute of Microbiology ETH-Zurich, Zurich, Switzerland

## 

Memory T and B lymphocytes and long lived plasma cells represent a repository of the antigenic experience of an individual. By analyzing the specificity and function of these cells we can gain insights into the human immune response and identify mechanisms of protection and immunopathology. We have developed methods to dissect the functional heterogeneity and antigenic repertoire of human T cells, B cells and plasma cells. These methods are used: i) to identify subsets of effector and memory T cells with distinct roles in immune surveillance and protection in different tissues against different classes of pathogens, and ii) to dissect the relative role of plasma cells and memory B cells in the antibody response to pathogens and to isolate broadly neutralizing antibodies. A better understanding of the class and specificity of the human immune response will be instrumental to guide the design of effective vaccines.

